# Phenotypic and WGS-derived antibiotic resistance patterns of *Salmonella* Enteritidis isolates from retail meat and environment during 2014 to 2019 in China

**DOI:** 10.3389/fmicb.2025.1502138

**Published:** 2025-01-27

**Authors:** Liya Zheng, Qiannan Di, Xuebin Xu, Liyuan Liu, Chunbo Qu, Phil Bremer, Xiujuan Zhou

**Affiliations:** ^1^College of Public Health, Shanghai University of Medicine and Health Sciences, Shanghai, China; ^2^Shanghai Center for Disease Control and Prevention, Shanghai, China; ^3^Department of Food Science, University of Otago, Dunedin, New Zealand; ^4^New Zealand Food Safety Science and Research Centre, Palmerston North, New Zealand

**Keywords:** *Salmonella* Enteritidis, whole-genome-sequencing, multidrug-resistant, clustering analysis, genetic determinants

## Abstract

The emergence of multidrug-resistant (MDR) *Salmonella* Enteritidis has highlighted the importance of regularly monitoring for the occurrence of antibiotic-resistant strains. The current study combined phenotyping analysis and whole-genome-sequencing (WGS) to investigate the associations between the antibiotic-resistant phenotypes (ARPs) and genetic characteristics determinants in 95 *Salmonella* Enteritidis isolates from retail meat and environmental samples in China (2014–2019). Phenotypic analyses revealed that 70 isolates (73.68%) were MDR with 12 distinct resistance patterns. Most MDR strains (81.43%) had NAL-AMP-FIS-STR ± TET profiles, showing a fluctuating trend from 2015 to 2019, likely influenced by tetracycline withdrawal management. WGS identified four types of mutations in the *gyrA* gene were associated with nalidixic acid resistance. The co-carrying of *bla*_TEM_, *sul2* and *aph(6)-Id/aph(3″)-Ib* was likely mediated by an X1-type plasmid, corresponding to resistance against ampicillin, sulfisoxazole, and streptomycin. Combining phenotypic analyses and WGS data, the 31 sequenced strains were primarily divided into two clusters, with most epidemic resistant strains in the largest cluster A. Identical ARP patterns observed across different sample types, regions, and isolation years but clustering together in cluster A suggested potential cross-contamination within the retail chain. Cluster B exhibited more diverse resistance patterns and genetic characteristics. Notably, three isolates in cluster B require special mention: a monophasic strain resistant to eight antibiotics, a strain exhibiting highly heteroresistance, and a strain with additional exotoxin genes. These results highlight the importance of ongoing surveillance and the utility of WGS to track and understand antibiotic resistance in *Salmonella* Enteritidis.

## 1 Introduction

Foodborne *Salmonella* Enteritidis is predominantly associated with eggs, chicken and related products (Hofer, 2021; Gu et al., 2020). However, this bacterium can occur throughout the food production chain, infecting other food animals and contaminating a wide range of foods (Guerrero et al., 2022; Bellil et al., 2023; Yang et al., 2020). *Salmonella* Enteritidis accounts for approximately 40–60% of global salmonellosis cases (Pearce et al., 2018) and is the most frequently detected serovar among patients with diarrhea in China (Wang et al., 2022). The main approach for treating salmonellosis relies on antibiotics, with third-generation cephalosporins and fluoroquinolones being the first-line clinical options (Konyali et al., 2023). However, due to the widespread use of antibiotics, the incidence of multi-drug resistant (MDR) *Salmonella* stains is increasing, with over 70% of isolates from patients with diarrhea, food animals, or retailed foods in China being reported as being MDR (Wang et al., 2020; Song et al., 2020). Previous investigations into *Salmonella* Enteritidis and other *Salmonella* serovars has demonstrated significant variations in the prevalence of antibiotic resistance among isolates from different sample types, geographic regions and years of isolation (Zakaria et al., 2022; Ksibi et al., 2022; Kang et al., 2022). Moreover, the antibiotic resistance patterns of this serovar often differ from those observed in other *Salmonella* serovars (Gu et al., 2020; Kang et al., 2022). Therefore, it is essential to continually update data on the resistance patterns of this prominent serovar across different time frames, regions and sample types to trace the origin of resistant strains and elucidate the development of their resistance profiles.

Antibiotic resistance in *Salmonella* can develop through point mutations in the bacterial genome or *via* the horizontal transfer of genetic elements carrying antibiotic resistant genes (ARGs) (Rakitin et al., 2021; Zhao et al., 2023). Whole genome sequencing (WGS) has become both a complementary and alternative approach to traditional methods for evaluating the genetic diversity of ARGs in *Salmonella* (Hu et al., 2020; Alzahrani et al., 2023). Additionally, WGS can provide valuable insights into other factors, such as virulence determinants, mobile genetic elements, and other genomic changes linked to pathogenicity and antibiotic resistance (Ksibi et al., 2022). Furthermore, WGS offers superior resolution and accuracy for correlation analyses based on the core genome compared to conventional molecular typing methods, such as pulsed-field gel electrophoresis (PFGE) (Edirmanasinghe et al., 2017; Keefer et al., 2019; Rounds et al., 2020). An enhanced ability to connect resistance and virulence phenotypes with genotypes, will facilitate epidemiological investigations, as detailed data on the genomic context of each isolate will help in the determination potential transmission routes (Edirmanasinghe et al., 2017; Sun et al., 2024; García-Soto et al., 2023).

The current study determined the occurrence of antibiotic resistance in 95 *Salmonella* Enteritidis isolates, primarily obtained from retail meat and environmental samples during 2014–2019 in China, and investigated the temporal and geographical distribution of antibiotic resistance patterns among these isolates. WGS analyses were conducted to determine the diversity of resistance and virulence genes and to analyze the genetic relatedness among these isolates. The findings provide a scientific basis for quantitatively assessing the public health risk posed by *Salmonella* Enteritidis.

## 2 Materials and methods

### 2.1 *Salmonella* Enteritidis isolates

A total of 95 *Salmonella* Enteritidis isolates were used in the study (Supplementary Table S1). Among the 95 isolates, 67 were isolated from chicken meat, 13 from duck meat, 5 from egg products, 4 from pork, 2 from river water environment, 2 from freshwater fish, and 1 from a food poisoning incident. Geographically, 48 of the isolates originated from Shanghai and Shandong in East China, 33 from Guangdong in South China, and 14 from other regions and cities across China. These isolates were collected over the period from 2014 to 2019. More detailed information of these isolates is listed in Supplementary Table S1. These isolates were stored in a − 80°C freezer in our laboratory and reactivated by overnight cultivation at 37°C prior to use. The serovar of every isolate was re-verified using a PCR method, as described in a previous study (Liu et al., 2012). In brief, the serotype-specific primer sen-1392 (Liu et al., 2012) for *Salmonella* Enteritidis was used for PCR, with slight adjustments to the PCR conditions. Specifically, the conditions included an initial denaturation at 94°C for 5 min, followed by 35 cycles of 94°C for 30 s, 60°C for 30 s, and 72°C for 30 s, with a final extension at 72°C for 10 min, and a final hold at 4°C. In the subsequent agarose gel electrophoresis, a 656 bp band was detected in the PCR products of all isolates, indicating that they were all *Salmonella* Enteritidis.

### 2.2 Antimicrobial susceptibility testing

According to the guidelines recommended by the Clinical and Laboratory Standards Institute (CLSI) (CLSI, 2020), the minimum inhibitory concentration (MIC) for a range of antibiotics was determined using the agar dilution method. A total of 14 antibiotics were tested, including amikacin (AMK), gentamicin (GEN), kanamycin (KAN), streptomycin (STR), ampicillin (AMP), ceftriaxone (CRO), cefepime (FEP), sulfisoxazole (FIS), chloramphenicol (CHL), tetracycline (TET), ciprofloxacin (CIP), ofloxacin (OFX), nalidixic (NAL) and fosfomycin (FOS). Bacterial cultures were prepared by inoculating fresh colonies into Mueller Hinton broth (Beijing Landbridge Technology Co., Ltd., Beijing, China) and incubating at 37°C for 18–24 h. The inoculum density was adjusted to the McFarland 0.5 standard prior to testing. The agar dilution method was performed by preparing antibiotic-containing Mueller Hinton agar (Beijing Landbridge Technology Co., Ltd., Beijing, China) plates in accordance with CLSI guidelines (CLSI, 2020), and the inoculated plates were incubated at 37°C for 18–24 h. *Escherichia coli* ATCC 25922 and *Staphylococcus aureus* ATCC 25923 were used as quality control bacteria in the MIC determinations. Breakpoints for all the tested antibiotics were used according to the interpretive standards by CLSI (2020). Bar charts for analysis on the distribution of sample types, geographic regions and years of isolation across different antibiotic resistance phenotypes (ARPs) were created using ChiPlot.^1^

### 2.3 Whole genome sequencing and clustering tree construction

Of the 95 isolates, 31 were selected for WGS based on their resistance profiles, sample types, isolation dates, and geographic distribution to ensure representation of the broader dataset. WGS was carried out by Illumina platform Hiseq 2,500 in the Majorbio Corporation (Shanghai, China). Briefly, genomic DNA was extracted from each strain using the TIAN amp Bacterial DNA Kit (Tiangen, Beijing, China) following the manufacturer’s instructions. All genomes were constructed as fragments with an insert length of 500 bp to generate sequencing libraries using the NEB Next Ultra DNA library Prey Kit for Illumina (NEB, Beverly, MA, USA) according to the manufacturer’s recommendations. All genomes were assembled from scratch using SPAdes.^2^ The core genomes were aligned using MAFFT.^3^ To infer the clustering relationships among the bacterial isolates based on their core genomes, maximum-likelihood (ML) trees were constructed using IQ-TREE.^4^ Clustering trees were visualized using FigTree^5^ and edited for clarity in Adobe Illustrator. In Adobe Illustrator, the tree images were adjusted by repositioning branches, resizing labels, and adding color to improve readability and enhance the visual presentation of the clustering relationships.

### 2.4 *In silico* analysis of genetic information and plasmid typing

The detection of ARGs and point mutations was accomplished using ResFinder.^6^ The virulence factors (VFs) were investigated through the Virulence Factor Database (VFDB).^7^ A threshold of 90% was established to define a significant match between the identified gene sequences and those known to confer resistance and virulence, while a minimum length coverage criterion of 60% was applied to further validate the presence of these ARGs and VFs in the isolates examined. The PlasmidFinder database^8^ was employed to detect and characterize plasmid replicons in the bacterial samples. The 90% nucleotide identity cutoff was used to filter out the corresponding plasmid replication types in each isolate. All these bioinformatics analyses were based on the 31 sequenced representative isolates.

## 3 Results

### 3.1 Antibiotic resistance of 95 *Salmonella* Enteritidis isolates

Among the 95 *Salmonella* Enteritidis isolates analyzed, resistance was most frequently observed for NAL (91/95, 95.79%), followed by AMP (75/95, 78.95%), FIS (65/95, 68.42%), STR (57/95, 60.00%), and TET (28/95, 29.47%). All isolates were susceptible to AMK, CIP, and OFX, with low resistance rates (ranging from 1.05 to 3.16%) to other tested agents including GEN (1/95, 1.05%), CHL (1/95, 1.05%), CRO (1/95, 1.05%), EFP (2/95, 2.11%), KAN (3/95, 3.16%), and FOS (3/95, 3.16%). Notably, most isolates exhibited low MICs to third-generation cephalosporins (CRO and EFP) and fluoroquinolones (CIP and OFX), with MIC90 values below 0.125 μg/mL (Table 1).

**Table 1:** Minimal inhibitory concentrations (MICs) among 95 *Salmonella* Enteritidis isolates.

Antibiotic agents	Distribution (no.) of MICs (μg/ml) among the 95 isolates																
	≤0.125	0.25	0.5	1	2	4	8	16	32	64	128	256	≥512	MIC50	MIC90	Resistant breakpoint	Resistance% (no.)
Amikacin (AMK)	95													≤0.125	≤0.125	≥64	0
Gentamicin (GEN)						94					1			4	4	≥16	1.05% (1/95)
Kanamycin (KAN)								92			1	1	1	16	16	≥64	3.16% (3/95)
Streptomycin (STR)								36	2	1	0	14	42	256	≥512	≥64	60.00% (57/95)
Ampicillin (AMP)							3	16	1	0	75			128	128	≥64	78.95% (75/95)
Ceftriaxone (CRO)	94							1						≤0.125	≤0.125	≥4	1.05% (1/95)
Cefepime (FEP)	93							2						≤0.125	≤0.125	≥16	2.11% (2/95)
Sulfisoxazole (FIS)											12	18	65	≥512	≥512	≥512	68.42% (65/95)
Chloramphenicol (CHL)							94				1			8	8	≥32	1.05% (1/95)
Tetracycline (TET)						66	1	2	1	24	1			4	64	≥16	29.47% (28/95)
Ciprofloxacin (CIP)	95													≤0.125	≤0.125	≥1	0
Ofloxacin (OFX)	95													≤0.125	≤0.125	≥8	0
Nalidixic acid (NAL)							4			1	3	87		256	256	≥32	95.79% (91/95)
Fosfomycin (FOS)										92			3	64	64	≥128	3.16% (3/95)

A total of 70 isolates (73.68%) were classified as MDR, defined as being resistant to three or more antibiotic agents. These isolates displayed 12 distinct MDR patterns (Table 2), including 1 isolate that showed resistance to 8 out of 14 antibiotics tested (NAL-AMP-FIS-STR-TET-KAN-FEP-CHL), with the underlined antibiotics forming the ACSSuT resistance pattern. The two most prevalent MDR profiles in this study were NAL-AMP-FIS-STR which was exhibited by 33 isolates and NAL-AMP-FIS-STR-TET which was exhibited by 24 isolates (Table 2). A third pattern (NAL-AMP-FIS) was exhibited by 3 isolates, and a fourth pattern (NAL-AMP-FIS-STR-FOS) by 2 isolates. Each of the other eight patterns was represented by a single isolate (Table 2). Along with 2 pan-sensitive strains, the remaining 23 strains which were resistant to one or two antibiotics were categorized into 5 different resistance profiles, with NAL resistance observed in 10 strains, and NAL-AMP resistance in 8 strains (Table 2).

**Table 2:** Antibiotic-resistant phenotype patterns among the 95 *Salmonella* Enteritidis isolates.

Antibiotic-resistant phenotypes	Prevalence, % (no.)
NAL-AMP-FIS-STR-TET-KAN-FEP-CHL	1.05% (1)
NAL-AMP-FIS-STR-TET	25.26% (24)
NAL-AMP-FIS-STR-FOS	2.11% (2)
NAL-AMP-STR-KAN-GEN	1.05% (1)
NAL-AMP-FIS-STR	34.74% (33)
NAL-AMP-TET-CRO	1.05% (1)
NAL-AMP-FIS	3.16% (3)
NAL-FIS-STR	1.05% (1)
NAL-STR-TET	1.05% (1)
NAL-STR-KAN	1.05% (1)
NAL-TET-FEP	1.05% (1)
AMP-FIS-STR	1.05% (1)
NAL-AMP	8.42% (8)
NAL-FIS	2.11% (2)
NAL-FOS	1.05% (1)
NAL	10.53% (10)
AMP	1.05% (1)
Pan-susceptive	2.11% (2)

AMP, ampicillin; NAL, Nalidixic acid; FEP, Cefepime; CRO, ceftriaxone; TET, tetracycline; STR, streptomycin; FIS, Sulfisoxazole; CHL, Chloramphenicol; GEN, Gentamicin; KAN, Kanamycin; FOS, Fosfomycin.

### 3.2 Distribution of sample sources across different ARPs

**Figure 1:**
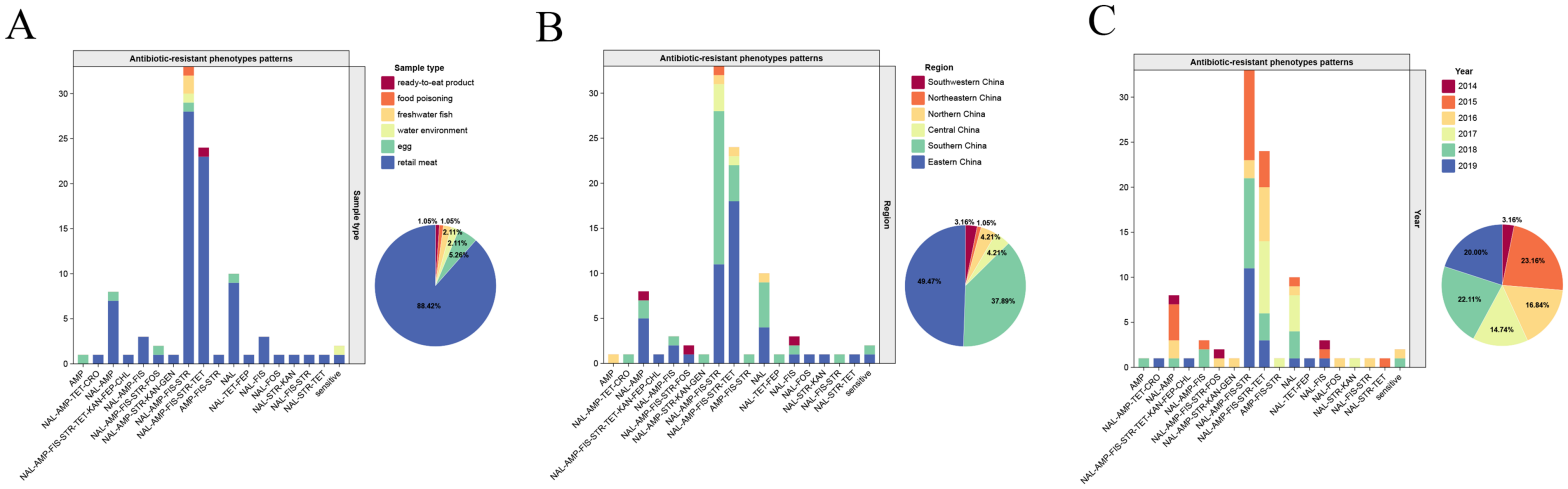
Distribution of sample types **(A)**, geographic regions **(B)** and years of isolation **(C)** across different ARPs. Each bar represents a specific sample type, region and year, and its height corresponds to the number of samples that exhibit a certain ARP. The meanings of the abbreviations in the antibiotic-resistant phenotype patterns were shown in Table 1. The pie chart in each subgraph represents the proportion of strains in different categories.

Bar charts were constructed to show the distribution of various sample types across different ARPs (Figure 1A). Retail meat samples exhibited almost all of the ARPs, with the phenotype AMP being the sole exception. The only strain from a food poisoning sample and two strains from freshwater fish all exhibited the phenotype NAL-AMP-FIS-STR, while the only strain from a ready-to-eat food sample exhibited the phenotype NAL-AMP-FIS-STR-TET. Both of these phenotypes were the top two most prevalent antibiotic resistance patterns in this study. Five strains from egg samples exhibited diverse ARPs. Among them, two strains were MDR: one exhibited the most prevalent resistance pattern (NAL-AMP-FIS-STR), while the other was also exhibited resistance to FOS. The remaining three strains were not extensively resistant, with each showing resistance to either AMP, NAL, or both of these antibiotics. Two strains from the river water environment showed significantly different ARPs to each other. One strain was fully sensitive to all the antibiotics tested, while the other stain exhibited the most prevalent resistance pattern (NAL-AMP-FIS-STR).

From the perspective of geographic regions (Figure 1B), the three isolates from Southwestern China did not belong to either of the two most prevalent resistant patterns. The sole isolate from Northeastern China exhibited the most prevalent resistant pattern. The two most prevalent resistant patterns were also both detected in the other four regions of China. From the perspective of isolation years (Figure 1C), the three strains from 2014 did not belong to either of the two most prevalent resistant patterns, and interestingly, they were from Southwestern China. The most prevalent resistant pattern was not detected in isolates from 2017, while the second most prevalent resistant pattern was detected in isolates from 2015 to 2019. The annual distribution of the two most prevalent resistance patterns (NAL-AMP-FIS-STR ± TET) varied considerably from year to year (Figure 1C), meaning that in some years (2016 and 2017), strains resistant to TET were more common, while in others (2015, 2018 and 2019), strains lacking TET resistance were more prevalent.

### 3.3 Genetic analysis of 31 representative *Salmonella* Enteritidis isolates

A total of 31 *Salmonella* Enteritidis isolates were selected as representative strains for WGS. These representative strains encompassed all observed ARPs and two sensitive strains. Strains were selected based on a combination of ARPs, sample types, regions, and years. If a particular phenotype was only identified once in the 95 isolates studied, it was also included in the selection.

Using the ResFinder online prediction tool, a total of 16 antibiotic resistance genes were detected. These genes potentially corresponded to six different resistance categories (Table 3) which are described in detail below.

**Table 3:** Prevalence of ARGs in diverse antibiotic-resistant categories for 31 sequenced strains.

Categories	Genotypes		Phenotypes		Compliance rate (%)
	ARGs	No. and prevalence (%)	Antibiotics	No.	
β-lactamase	*bla*_TEM_	24 (77.42%)	AMP	23	95.83%
	*bla*_CTX-M-55_	1 (3.23%)	FEP	1	100%
Aminoglycosides	*aac(6′)-Iaa*	31 (100.00%)	NCP	NCP	NCP
	*aph(6)-Id*	20 (64.52%)	STR	19	95%
	*aph(3″)-Ib*	20 (64.52%)	STR	19	95%
	*aph(3′)-IIa*	4 (12.90%)	STR/KAN/GEN	4/2/1	100%/50%/25%
	*aac(3)-IId*	3 (9.68%)	STR/KAN/GEN	3/1/1	100%/33.3%/33.3%
	*aadA5*	1 (3.23%)	NCP	NCP	NCP
Chloramphenicol	*floR*	1 (3.23%)	CHL	1	100%
Tetracycline	*tet(A)*	6 (19.35%)	TET	5	83.33%
Fosfomycin	*fosB*	1 (3.23%)	FOS	0	0
Sulfonamide	*dfrA17*	1 (3.23%)	NCP	NCP	NCP
	*sul2*	20 (64.52%)	FIS	20	100%

NCP, no corresponding phenotype was found; *Compliance rate means the proportion of genes with corresponding phenotypes out of the total number of detected genes.

#### 3.3.1 Aminoglycoside resistance genes

**Figure 2:**
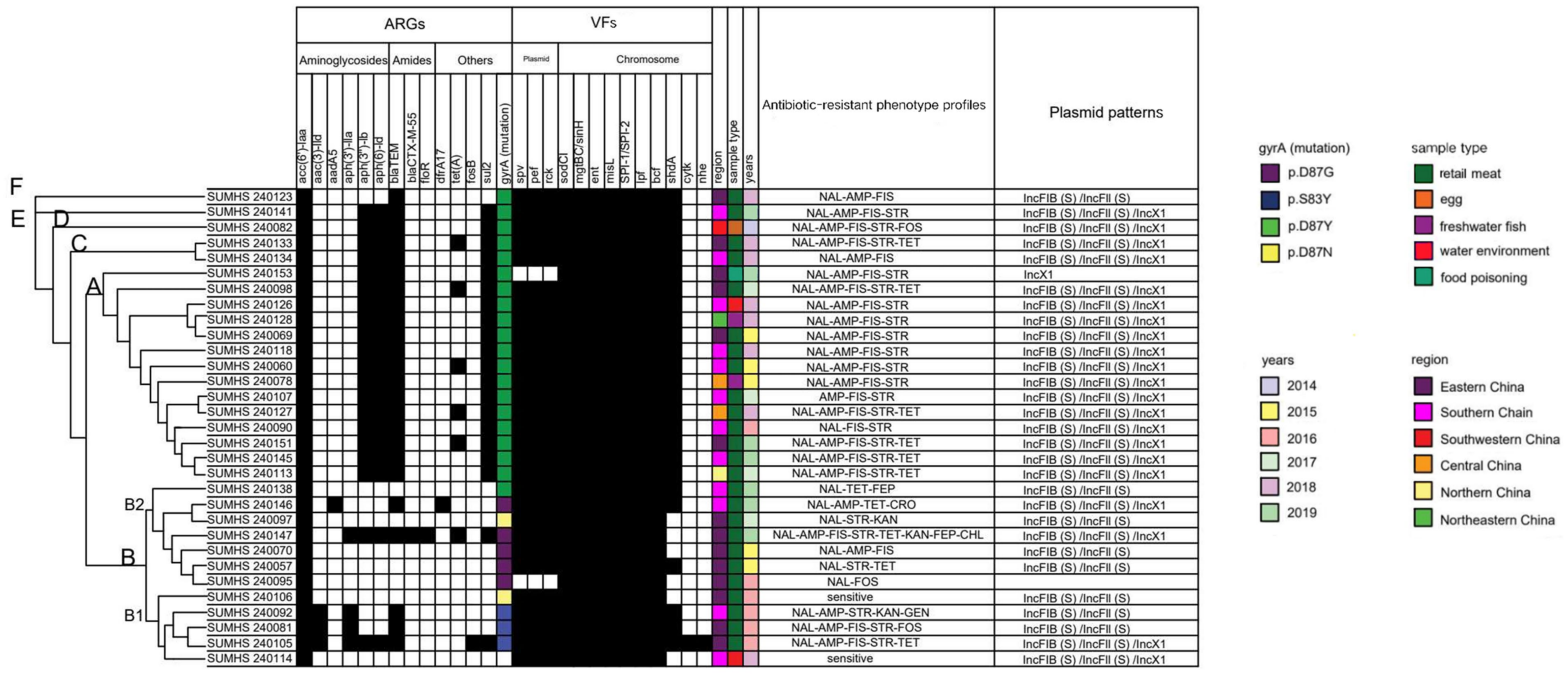
Clustering tree based on core genome and heatmap of 31 *Salmonella* Enteritidis strains: in silico analysis of virulence factors (VFs), antibiotic resistance genes (ARGs) and plasmid pattens.

Six aminoglycoside resistance related genes were identified among the isolates (Table 3 and Figure 2). The three most prevalent genes were *aac(6′)-Iaa*, *aph(6)-Id*, and *aph(3″)-Ib*, which were detected in 100% (31/31), 64.52% (20/31), and 64.52% (20/31) of the isolates, respectively. Additionally, the *aph(3′)-IIa* gene was present in 12.90% (4/31) of the isolates, the *aac(3)-IId* gene was found in 9.68% (3/31), and the *aadA5* gene was identified in 3.23% (1/31) of the isolates. The simultaneous or partial presence of the genes *aph(6)-Id*, *aph(3″)-Ib*, *aph(3′)-IIa*, and *aac(3)-IId* was linked to the STR phenotype, with compliance rates of 95 to 100% (Table 3). Additionally, the latter two genes (*aph(3′)-IIa* and *aac(3)-IId*) were also associated with the aminoglycosides GEN and KAN to some extent (Table 3).

#### 3.3.2 *β*-lactam resistance genes

Two distinct β-lactam resistance genes were identified (Table 3 and Figure 2). The *bla*_TEM_ gene was present in 77.42% (24/31) of the isolates, most of which were associated with AMP, with the compliance rate at 95.83% (Table 3). The *bla*_CTX-M-55_ gene was detected in only 1 isolate (3.23%) (SUMHS 240147), which also exhibited resistance to FEP (Table 3 and Figure 2).

#### 3.3.3 Quinolone resistance genes

Resistance to the quinolone antibiotic, NAL, was primarily linked to chromosomal mutations in the DNA gyrase subunit gene (*gyrA*). The mutation in *gyrA* gene was identified in 30 out of 31 isolates that classified into 4 types. The predominant mutation site was *gyrA* [87:D-Y] (66.67%, 20/30), followed by *gyrA* [87:D-G] (16.67%, 5/30), *gyrA* [83:S-Y] (10%, 3/30), and *gyrA* [87:D-N] (6.67%, 2/30) (Figure 2).

#### 3.3.4 Other resistance genes

One or two resistance genes were identified in each of the following antibiotic resistance groups: TET (*tetA* gene), sulfonamides (*sul2* and *dfrA17* genes), CHL (*floR* gene) and FOS (*fosB* gene). The *sul2* gene was found in 20/31 (64.52%) isolatess, while the *tetA* gene was detected in 6/31 (19.35%) isolate. The *dfrA17* gene was identified in isolate SUMHS 240146, and the *floR* gene was found in isolate SUMHS 240147, in addition the *fosB* gene was identified in isolate SUMHS 240105 (Figure 2). The ARPs of most isolates generally aligned with their corresponding ARGs, such as *floR* to CHL, and *sul2* to FIS, which had the 100% compliance rate (Table 3). However, some isolates exhibited resistance phenotypes for which a genetic basis could not be identified, while in other cases, ARGs were present in isolates that remained susceptible to the corresponding antibiotics. For example, although isolate SUMHS 240105 harbored the *fosB* gene, it did not show resistance to FOS; while three FOS resistance strains did not contain the *fosB* gene, which suggested that in these isolates resistance to FOS was due to yet to be identified gene(s). Similarly, six isolates were resistant to TET but lacked the *tet(A)* gene, which is typically responsible for TET resistance. Notably, among the 31 isolates, those (20/31) carrying the *aph(6)-Id* and *aph(3″)-Ib* genes were also found to possess the *sul2* gene, and the *bla*_TEM_ gene, associated with STR resistance, was frequently found in combination with both FIS and AMP (Figure 2).

Similarly, virulence genes and plasmid replicon types among the 31 isolates were detected using the VFDB online prediction tool and the PlasmidFinder database, respectively.

#### 3.3.5 Virulence factors

Type III secretion systems (T3SS) encoded by *Salmonella* Pathogenicity Island (SPI-1 and SPI-2) were present in all 31 *Salmonella* Enteritidis isolates. The superoxide dismutase gene, *sodC1*, and fimbria related gene *lpf*, along with the metal ion-regulating genes *mgtBC* and *misL*, were also found in 100% of the isolates (Figure 2). Another fimbria related gene *shdA* was present in 80.65% (25/31) of the isolates. Additionally, plasmid mediated virulence genes were detected across most of strains (93.5%, 29/31), including fimbrial adhesins (*pef*), T3SS effectors (*spv*), and the complement resistance gene (*rck*). The remaining two virulence genes were less common, with *nhe* and *cytK* only being present in 3.23% (1/31) of the strains (Figure 2).

#### 3.3.6 Plasmids assays

The plasmid profiles indicated that F-type (IncFIB/IncFII) and X1-type plasmids were common in *Salmonella* Enteritidis strains (Figure 2). F-type plasmids were present in 93.55% (29/31) of the strains, while X1-type plasmids were detected in 64.52% (20/31) of the strains. No plasmids were identified in SUMHS 240095, whereas only the X1-type plasmid was detected in SUMHS 240153. Notably, the presence of the X1-type plasmid corresponded with the co-occurrence of *bla*_TEM_, *sul2* and *aph(6)-Id*/*aph(3″)-Ib*, as mentioned above, suggesting a high likelihood that these genes were located on the X1-type plasmid, conferring resistance to AMP-FIS-STR.

### 3.4 Genetic diversity and relatedness of 31 *Salmonella* Enteritidis isolates

For the purpose of illustrating the interrelationships among these *Salmonella* Enteritidis isolates, a clustering tree was constructed based on the core genome genes (Figure 2). This classified tree partitioned the 31 *Salmonella* Enteritidis isolates into 6 distinct clusters. Predominantly, two larger clusters were identified: cluster A, which encompassed 14 isolates, and cluster B, which was comprised of 12 isolates. Cluster B was distinctly subdivisible into B1, which encompasses 5 isolates, and B2, which contained the remaining 7 isolates. The remaining clusters, designated C, D, E and F, were notably smaller, each incorporating only 1 or 2 isolates.

The majority (7/8) of isolates carrying the most prevalent ARP (NAL-AMP-FIS-STR) (Figure 2) were classified into cluster A (Figure 2), which exhibited similar resistance and virulence genes, suggesting clonal transmission. However, the sample types, regions, and years of these 7 isolates differed, suggesting that strains with this ARP may be widespread in retail markets and the environment. This indicates that clonal transmission may be occurring due to cross-contamination during the sales process or food chain, likely involving the spread of bacteria between products, surfaces, or handlers, leading to the same bacterial clone being present in different locations or at different times. Notably, 6 closely clustered isolates were from 2015 to 2018, with the other 2 isolates, from 2019, having slightly longer clustering distances, which indicates that the genomes of the most recent isolates have slightly changed. Taking strain SUMHS 240153 as an example, the absence of three virulence genes, *spv*, *pef* and *rcs*, typically found on the virulence plasmid, was observed. This suggests that the strain might have diverged from the other 6 strains due to the potential loss of this specific virulence plasmid after infecting humans, as this was a food poisoning isolate. Similarly, the majority (5/7) of the isolates that possessed the second most prevalent ARP (NAL-AMP-FIS-STR-TET) (Table 2) were also classified into cluster A (Figure 2). These stains while all being isolated from retail meat, came from different regions and were isolated between 2017 and 2019, indicating that this prevalent resistance strain was most likely clonally transmitted through retail meat in various regions. Notably, one isolate (SUMHS 240105) exhibiting the second most prevalent ARP was classified into cluster B. This isolate differed significantly from the above 5 strains in terms of the resistance and virulence genes detected. For instance, it lacked the *tet* gene but exhibits a TET phenotype; 4 additional resistance genes [*aac(3)-IId*, *aph(3″)-Ib*, *aph(3′)-IIa*, *fosB*] were detected, yet they had no corresponding resistant phenotypes, referred to as heteroresistance; additionally, 2 virulence genes (*ctyK* and *nhe*) were uniquely found within this strain and the type of mutation in *gyrA* (S83Y) was different from others in cluster A. All of this data suggests that this strain may be a completely different clone.

Overall, both the ARGs and ARPs were more complicated in cluster B compared to for the other 5 clusters. Particularly, cluster B included four types of *gyrA* gene mutants (D87Y, D87N, D87G, and S83Y), whereas only the most typical mutation (*gyrA*: D87Y) existed in the other clusters (A, C, D, E, F). Similarly, the carriage of ARGs in the aminoglycoside class were uniform in the other 4 clusters (A, C, D, E, F), whereas this was not the case in cluster B. Additionally, the only two pan-susceptible strains among the tested isolates were also located within the B1 sub-branch of cluster B. With regard to geographic distribution and sample types; almost all the isolates in cluster B were from retail meat and all of them were isolated from Eastern or Southern China between 2015 and 2019. This suggested that these isolates in cluster B may have a wealth of transferable components which are carrying different ARGs or virulence genes.

## 4 Discussion

The current study analyzed the antibiotic resistance profiles of *Salmonella* Enteritidis isolates in China from 2014 to 2019 (Table 1 and Figure 1). Compared to data reported for *Salmonella* Enteritidis strains isolated from retail chicken samples from Shanghai between 2008 and 2012 (Zhou et al., 2018), resistance rates appear to have increased significantly: AMP from 50.70 to 78.95%, FIS from 49.32 to 68.42%, TET from 17.12 to 29.47%, and STR from 4.80% to a notable 60.00%. Further, compared to *Salmonella* Enteritidis strains isolated from clinical samples in Beijing between 2010 and 2014 (Qu et al., 2016), resistance rates also showed slight increases: AMP increased from 60.0 to 78.95%, FIS from 54.3 to 68.42%, and STR from 42.9 to 60.00%. These findings indicate a worrying increase in the resistance of *Salmonella* Enteritidis to commonly used antibiotics, which if not checked could limit treatment options and make infections harder to manage. Fortunately, current monitoring data from retail samples of *Salmonella* Enteritidis indicate that resistance to first-line clinical antibiotics, such as cephalosporins and fluoroquinolones, remains low (Yang et al., 2020; Zhou et al., 2018; Yin et al., 2022; Kanaan et al., 2023). The results of the current study support these observations, as all isolates were sensitive to fluoroquinolones and resistance to cephalosporins was limited, with the MIC90 values for both antibiotics being comparatively low (Table 1). In contrast, the situation is less optimistic in clinical settings for *Salmonella* Enteritidis (Li et al., 2021), as well as for other *Salmonella* serovars in retail samples, such as *Salmonella Typhimurium* (Yang et al., 2023) and *Salmonella* Indiana (Hu et al., 2022).

Although 12 different MDR profiles were identified in this study (Table 2), the majority of isolates (81.43%, 57/70) exhibited the NAL-AMP-FIS-STR ± TET profiles (Table 2 and Figure 1), which was consistent with the predominant resistance pattern reported for *Salmonella* Enteritidis in retail foods in Shanghai in 2020 (Yang et al., 2020) and in Shaanxi in 2021 (Dai et al., 2021). Interesting, the relative prevalence of the two most common resistance profiles differed from 2015 to 2019, owing to the presence or absence of TET (Figure 1C). TET was once widely used to promote growth and prevent animal diseases (Liang et al., 2023; Rincón-Gamboa et al., 2021). However, its non-therapeutic use, in China was strictly regulated during the years the isolates in the current study were obtained from, particularly in food products like meat, eggs, and milk, with strict adherence to withdrawal periods (Xu et al., 2020). This regulation may in part explain the alternating patterns of *Salmonella* Enteritidis resistance to TET and underscore the link between antibiotic resistance in retail meat strains and antibiotic use in farming. With new policies prohibiting TET as a growth-promoting feed additive since 2020, resistance to this antibiotic is expected to decline.

In the current study, a notable finding was the identification of strain SUMHS 240147, isolated from retail chicken in Shanghai in 2019, which exhibited resistance to eight antibiotics (Figure 2) and may be a derivative of the ACSSuT resistance pattern. The ACSSuT profile is typically associated with the main MDR type in *Salmonella Typhimurium* (Yang et al., 2020) but is rarely found in *Salmonella* Enteritidis. Recent studies have shown that the ACSSuT pattern is usually linked to high resistance against third-generation cephalosporins and quinolones in *Salmonella*, including Typhimurium (Yang et al., 2020), Newport and Dublin (Bhandari et al., 2023), and Enteritidis (Ma et al., 2018), which aligns with the findings of this study. These results highlight the need for regular monitoring to track changes in antibiotic resistance patterns, serving as a warning for other cities in China.

The egg-derived isolates in the current study exhibited five different ARP patterns, demonstrating a relatively rich resistance profile (Figure 1A). This is similar to a recent report on antibiotic resistance in *Salmonella* from poultry eggs (Bahramianfard et al., 2021), which showed that although the number of antibiotics *Salmonella* were resistant to was low, the resistance profiles were quite complex. A representative egg isolates (SUMHS 240082), which was sequenced was categorized into cluster D (Figure 2), exhibiting an additional FOS resistance compared to the second most prevalent resistant pattern (NAL-AMP-FIS-STR-TET). It shared the same resistance phenotype with a strain (SUMHS 240081) from the distantly related cluster B1, yet it had a significantly different genotype. These results suggested that the egg may not have been contaminated at the retail level and likely originated from chickens raised on farms that were infected with resistant *Salmonella* Enteritidis.

*In silico* analysis of antibiotic genes in the current study revealed complex relationships between the presence of specific genes and aminoglycoside phenotypes in *Salmonella* Enteritidis. While some genes, like *aph(3″)-Ib* and *aph(6)-Id*, showed a clear association with STR resistance, others, such as *aac(6′)-Iaa* and *aac(3)-IId*, did not demonstrate a clear correlation with resistance to certain aminoglycosides (Table 3). Previous studies have shown that the *aph(3″)-Ib* and *aph(6)-Id* genes are often detected together in STR-resistant strains (Srednik et al., 2022; Yue et al., 2022), which is consistent with the results obtained in the current study, where both genes were present in 20 out of 21 STR-resistant strains (concordance rate of 95%) (Table 3). Conversely, the gene *aac(3)-IId* which is sometimes associated with resistance to GEN (Davies et al., 2022; Cox et al., 2022) was detected in three strains of this study (Table 3 and Figure 2). However, among the three strains carrying the *aac(3)-IId* gene detected in this study, only one (SUMHS 240092) exhibited resistance to GEN (Figure 2). Furthermore, it was observed that for the aminoglycoside-resistant strains of the B cluster, the number of resistance genes did not correlate directly with the number of aminoglycoside resistance types (Figure 2). For instance, SUMHS 240105, which carried all four of the aforementioned genes, was only resistant to STR, while SUMHS 240092, which carried only *aac(6′)-Iaa* and *aac(3)-IId*, was resistant to STR, GEN, and KAN (Figure 2). This observation suggests that aminoglycoside resistance mechanisms in *Salmonella* Enteritidis are multifactorial and may involve additional factors beyond the presence and quantity of specific resistance genes. Further research is needed to fully elucidate these complexities.

Previous studies have indicated that *bla*_TEM_ is the most common gene associated with AMP resistance (Zheng et al., 2021). Accordingly, the current study observed a 95.83% concordance rate (23/24) between the presence of this gene and AMP resistance (Table 3). Additionally, an association of the IncX1 plasmid with the *bla*_TEM_ gene in *Salmonella* has previously been reported (Petrin et al., 2023). Consistent with this, 83.3% (20/24) of the AMP-resistant isolates in the current study carried the IncX1 plasmid (Figure 2), suggesting a high likelihood that the *bla*_TEM_ gene in *Salmonella* Enteritidis is carried by the IncX1 plasmid. Moreover, the co-occurrence of *bla*_TEM_, *sul2* and *aph(6)-Id*/*aph(3″)-Ib*, mediated by the X1 plasmid and resistance to AMP-FIS-STR, was also observed in a similar study by Li et al. (2022) for *Salmonella* isolated from dead poultry. This consistency suggests that the X1 plasmid plays a significant role in transmitting MDR among *Salmonella* strains from food animals to food products. Furthermore, the current study identified a strain (SUMHS 240147) carrying the *bla*_CTX-M-55_ gene that exhibited resistance to FEP (Figure 2). This finding aligns with previous reports that *bla*_CTX-M_ encodes a plasmid-mediated enzyme that preferentially hydrolyzes CRO or FEP in *Salmonella* (Long et al., 2022). Interestingly, the current study detected one FEP-resistant strain (SUMHS 240138) and one CRO-resistant strain (SUMHS 240146) that lacked *bla*_CTX-M_, suggesting the presence of other genes encoding broad-spectrum cephalosporin resistance that we did not detect. Similarly, the *tetA* gene, which is associated with phenotypic resistance to TET (Petrin et al., 2023), was found in only 6 out of 11 TET-resistant strains. As these genes are plasmid-mediated, discrepancies may arise from variations in plasmid abundance or sequencing accuracy. The *fosB* gene, commonly found in Gram-positive bacteria such as *Staphylococcus aureus* (Hu et al., 2023), is associated with FOS resistance (Lamers et al., 2012). However, it is rarely detected in Gram-negative bacteria, such as *Salmonella*, and its association with resistance in this context is uncertain. This observation might explain why one strain in the current study which carried the *fosB* gene did not exhibit FOS resistance. In *Salmonella*, FOS resistance is typically linked to mutations in chromosomal genes *glpT* and *murA* (Couce et al., 2012; Takahata et al., 2010) or plasmid-mediated genes like *fosA3* (Fang et al., 2020). However, the three FOS-resistant strains in the current study did not show the presence of these genes or mutations. This could be due to insufficient detection sensitivity or the involvement of other untested genes. Therefore, the aforementioned strains that exhibited resistance but lacked the corresponding genes, require more detailed sequencing and mechanistic studies.

Two specific strains classified in cluster B2 in the current study were also noteworthy. The strain SUMHS 240147 which exhibited an 8-fold resistant phenotype was isolated in 2019 (Figure 2), which was the most recent isolate among the batch of strains tested, making it worthy of attention at the current time. The ARPs were consistent with the ARGs, especially, with MDR-ACSSuT resistance commonly found on transferable components (e.g., plasmid) (Wottlin et al., 2022). However, MDR-ACSSuT resistance can also be located in chromosomes, as seen in *Salmonella Typhimurium*, where it is encoded within a pathogenic island (de Curraize et al., 2017). This suggests that the resistance might have originated from a common strain that underwent genetic mutation and acquired resistance genes. Interestingly, this strain was a monophasic variant (Supplementary Table S1). The emergence of such variants, which are frequently reported in *Salmonella Typhimurium* (Sun et al., 2020), could present certain challenges for the diagnosis and treatment of salmonellosis. Moreover, monophasic *Salmonella Typhimurium* often display increased resistance and pathogenicity (Sun et al., 2020; Qin et al., 2022). Therefore, this monophasic *Salmonella* Enteritidis strain, which exhibited high-quantity antibiotic resistance, warrants further toxicity testing. Another strain in cluster B2 that warrants attention was SUMHS 240146, which was extraordinarily unique in both phenotype and genotype. Specifically, it displayed resistance to CRO and TET, yet no corresponding ARGs were identified, indicating that it may have plasmid mediated unidentified genes. It was the only strain that carried the *dfrA17* and *aadA5* genes (Figure 2), which encode a dihydrofolate reductase enzyme for sulfamethoxazole/trimethoprim and an aminoglycoside-modifying enzyme (Ma et al., 2022), respectively. However, resistance to sulfonamides or aminoglycosides was not detected (Figure 2). Previous studies have shown that the *dfrA17*-*aadA5* gene cassette is often found together with the *aac(3)-Id* gene in type I integrons (Meng et al., 2017). However, this strain did not carry the *aac(3)-Id* gene, suggesting it may possess a defective type I integron that contains genes related to sulfonamides or aminoglycosides, but these genes are not expressed. These discrepancies between the WGS-determined genotype and experimentally observed ARP, referred to as heteroresistance, may limit the ability of WGS to accurately predict true resistance rates (Zwe et al., 2020). Heteroresistance includes false negative errors (where phenotypic resistance is absent despite the presence of the genetic resistance determinant) and false positive errors (where resistance genes are present without corresponding phenotypic resistance). Unstable, temporary changes in genetic elements may be the main reasons that cause heteroresistance (Zwe et al., 2020). Unstable genetic features such as temporary loss of gene expression or lower copy number when resistance genes are located on mobile elements could not be reliably employed by WGS to predict phenotype, especially in next-generation sequencing (NGS) platforms utilized in this study due to its short length of the reads generated. Therefore, incorporating deep sequencing specifically targeting these mobile elements may offer a potential solution to alleviate the detection failures *in silico*.

From a virulence perspective, these strains also have a high pathogenic potential. All these resistant isolates possessed relatively abundant virulence genes as genes related to antioxidant (*sodC1*), metal ion transport (*mgtBC* and *misL*), bacteriocin production (*ent*), and major pathogenicity islands (SPI1 and SPI2), as well as genes related to adhesion that may be located within the pathogenicity islands (*lpf* and *bcf*), were present in all the strains (Figure 2). Strangely, half (6/12) of the isolates classified into cluster B lacked the *shdA* gene, which encodes a protein involved in bacterial flagella synthesis and assembly (Urrutia et al., 2014), and it is usually not located in pathogenicity islands. Since the loss of this flagellar gene was only detected in monophasic bacteria in this study (Supplementary Table S1), it is hypothesized that there may be a relationship between the two, which requires further confirmation. Genes related to cytotoxins (*cytK*) and enterotoxins (*nhe*) were only detected in the SUMHS 240105 strain, which is also the only strain classified into cluster B among those with the second epidemic resistance pattern (NAL-AMP-FIS-STR-TET) (Table 2). Therefore, this highly virulent and epidemic resistant bacteria (SUMHS 240105) is also very worthy of further study because these exotoxins, including cytotoxins and enterotoxins, are believed to be responsible for diarrheal food poisoning (Stenfors Arnesen et al., 2008). In the current study, strains that did not harbor the virulence genes *pef*, *spv*, and *rck* also did not carry F-type plasmids, and vice versa (Figure 2). This confirmed previous reports that these three virulence genes are mediated by F-type virulence plasmids specific in *Salmonella* Enteritidis (Ksibi et al., 2022; Silva et al., 2017; Villa et al., 2010).

A limitation of this study is that WGS was conducted on only 31 of the 95 isolates due to resource constraints. While these isolates were selected to represent diverse resistance profiles, future studies should aim to sequence all isolates or conduct in-depth sequencing (e.g., nanopore sequencing) on the three isolates that warrant special attention and compare them with similar strains in public databases to provide a more comprehensive understanding of the genomic determinants and resistance mechanisms.

## 5 Conclusion

This analysis of antibiotic susceptibility among 95 *Salmonella* Enteritidis isolates revealed a notable rise in resistance to AMP, FIS, STR, and TET. Fortunately, the resistance rate for first-line antibiotics remained comparatively low. The yearly fluctuations in TET resistance may have been influenced by the management of withdrawal periods. These findings highlight the importance of continuous monitoring. The prevalence of ARPs across various sample types, regions, and isolation years suggests the potential for cross-contamination through the retail chain. However, the complex resistance profiles of egg-derived isolates indicated that contamination might have occurred at the animal or farm level. Leveraging WGS data, we identified most ARGs that align with observed ARPs, while variations in VFs were also uncovered. Co-carrying of *bla*_TEM_, *sul2* and *aph(6)-Id*/*aph(3″)-Ib (64.52%)* was likely mediated by an X1-type plasmid, while virulence genes including *pef*, *spv*, and *rck* were associated with an F-type plasmid. Clustering analysis, based on core genes from 31 representative strains, demonstrated the diversity in the development of resistance in *Salmonella* Enteritidis. Strains in cluster B exhibited distinct ARPs and ARGs. Notably, three strains in cluster B emerged with unique and potentially high-risk resistance phenotypes, as well as specific virulence and resistance genes, underscoring the need for vigilant monitoring of *Salmonella* Enteritidis. These findings provided new insights into the molecular epidemiology of antibiotic resistance and highlight critical intervention points for mitigating this public health threat.

## Data Availability

The complete sequences of 31 *Salmonella* Enteritidis have been deposited in the NCBI database 677 under PRJNA1161004.

## Footnotes

^1^ https://www.chiplot.online/

^2^ http://cab.spbu.ru/software/spades/

^3^ https://mafft.cbrc.jp/alignment/software

^4^ http://www.iqtree.org/

^5^ http://tree.bio.ed.ac.uk/software/figtree

^6^ https://cge.cbs.dtu.dk/services/ResFinder/

^7^ http://www.mgc.ac.cn/VFs/

^8^ https://cge.cbs.dtu.dk/services/PlasmidFinder/

## References

[ref1] Alzahrani K. O. Al-Reshoodi F. M. Alshdokhi E. A. Alhamed A. S. Al Hadlaq M. A. Mujallad M. I.. (2023). Antimicrobial resistance and genomic characterization of *Salmonella enterica* isolates from chicken meat. Front. Microbiol. 14:1104164. doi: 10.3389/fmicb.2023.1104164, PMID: 37065154 PMC10100587

[ref2] Bahramianfard H. Derakhshandeh A. Naziri Z. Khaltabadi Farahani R. (2021). Prevalence, virulence factor and antimicrobial resistance analysis of *Salmonella enteritidis* from poultry and egg samples in Iran. BMC Vet. Res. 17:196. doi: 10.1186/s12917-021-02900-2, PMID: 34030671 PMC8142639

[ref3] Bellil Z. Mairi A. Kendi S. Touati A. (2023). Nontyphoid *Salmonella* in farm animals and food products in the Middle East and North Africa: a systematic review. Future Microbiol. 18, 521–534. doi: 10.2217/fmb-2022-0239, PMID: 37309775

[ref4] Bhandari M. Poelstra J. W. Kauffman M. Varghese B. Helmy Y. A. Scaria J.. (2023). Genomic diversity, antimicrobial resistance, plasmidome, and virulence profiles of *Salmonella* isolated from small specialty crop farms revealed by whole-genome sequencing. Antibiotics. 12:1637. doi: 10.3390/antibiotics12111637, PMID: 37998839 PMC10668983

[ref5] CLSI (Ed.) (2020). Performance standards for antimicrobial susceptibility testing. 30th ed. CLSI supplement M100. Wayne, PA: Clinical and Laboratory Standards Institute.

[ref6] Couce A. Briales A. Rodríguez-Rojas A. Costas C. Pascual A. Blázquez J. (2012). Genome wide overexpression screen for fosfomycin resistance in *Escherichia coli*: MurA confers clinical resistance at low fitness cost. Antimicrob. Agents Chemother. 56, 2767–2769. doi: 10.1128/AAC.06122-11, PMID: 22371901 PMC3346583

[ref7] Cox G. W. Avery B. P. Parmley E. J. Irwin R. J. Reid-Smith R. J. Deckert A. E.. (2022). A one health genomic investigation of gentamicin resistance in *Escherichia coli* from human and chicken sources in Canada, 2014 to 2017. Antimicrob. Agents Chemother. 66:e0067722. doi: 10.1128/aac.00677-22, PMID: 36165686 PMC9578425

[ref8] Dai W. Zhang Y. Zhang J. Xue C. Yan J. Li X.. (2021). Analysis of antibiotic-induced drug resistance of *Salmonella enteritidis* and its biofilm formation mechanism. Bioengineered. 12, 10254–10263. doi: 10.1080/21655979.2021.1988251, PMID: 34637696 PMC8809914

[ref9] Davies N. Jørgensen F. Willis C. McLauchlin J. Chattaway M. A. (2022). Whole genome sequencing reveals antimicrobial resistance determinants (AMR genes) of *Salmonella enterica* recovered from raw chicken and ready-to-eat leaves imported into England between 2014 and 2019. J. Appl. Microbiol. 133, 2569–2582. doi: 10.1111/jam.15728, PMID: 35880358 PMC9804530

[ref10] de Curraize C. Amoureux L. Bador J. Chapuis A. Siebor E. Clément C.. (2017). Does the *Salmonella* Genomic Island 1 (SGI1) confer invasiveness properties to human isolates? BMC Infect. Dis. 17:741. doi: 10.1186/s12879-017-2847-1, PMID: 29195496 PMC5709944

[ref11] Edirmanasinghe R. Finley R. Parmley E. J. Avery B. P. Carson C. Bekal S.. (2017). A whole-genome sequencing approach to study cefoxitin-resistant *Salmonella enterica* serovar Heidelberg isolates from various sources. Antimicrob. Agents Chemother. 61, e01919–e01916. doi: 10.1128/AAC.01919-16, PMID: 28137797 PMC5365727

[ref12] Fang L. X. Jiang Q. Deng G. H. He B. Sun R. Y. Zhang J. F.. (2020). Diverse and flexible transmission of *fosA3* associated with heterogeneous multidrug resistance regions in *Salmonella enterica* serovar typhimurium and Indiana isolates. Antimicrob. Agents Chemother. 64, e02001–e02019. doi: 10.1128/AAC.02001-19, PMID: 31712202 PMC6985750

[ref13] García-Soto S. Linde J. Methner U. (2023). Epidemiological analysis on the occurrence of *Salmonella enterica* subspecies *enterica* serovar Dublin in the German federal state Schleswig-Holstein using whole-genome sequencing. Microorganisms 11:122. doi: 10.3390/microorganisms11010122 36677417 PMC9863307

[ref14] Gu D. Wang Z. Tian Y. Kang X. Meng C. Chen X.. (2020). Prevalence of *Salmonella* isolates and their distribution based on whole-genome sequence in a chicken slaughterhouse in Jiangsu, China. Front. Vet. Sci. 7:29. doi: 10.3389/fvets.2020.00029, PMID: 32154275 PMC7046563

[ref15] Guerrero T. Bayas-Rea R. Erazo E. Zapata Mena S. (2022). Nontyphoidal *Salmonella* in food from Latin America: a systematic review. Foodborne Pathog. Dis. 19, 85–103. doi: 10.1089/fpd.2020.2925, PMID: 34668752

[ref16] Hofer U. (2021). *Salmonella enteritidis*: chicken or egg? Nat. Rev. Microbiol. 19:682. doi: 10.1038/s41579-021-00632-6 34493850

[ref17] Hu J. Chen L. Li G. Pan Y. Lu Y. Chen J.. (2023). Prevalence and genetic characteristics of *fosB*-positive *Staphylococcus aureus* in duck farms in Guangdong, China in 2020. J. Antimicrob. Chemother. 78, 802–809. doi: 10.1093/jac/dkad014, PMID: 36691844

[ref18] Hu L. Cao G. Brown E. W. Allard M. W. Ma L. M. Khan A. A.. (2020). Antimicrobial resistance and related gene analysis of *Salmonella* from egg and chicken sources by whole-genome sequencing. Poult. Sci. 99, 7076–7083. doi: 10.1016/j.psj.2020.10.011, PMID: 33248624 PMC7705029

[ref19] Hu Y. He Y. Nguyen S. V. Liu C. Liu C. Gan X.. (2022). Antimicrobial resistance of *Salmonella* Indiana from retail chickens in China and emergence of an *mcr-1*-harboring isolate with concurrent resistance to ciprofloxacin, cefotaxime, and colistin. Front. Microbiol. 13:955827. doi: 10.3389/fmicb.2022.955827, PMID: 36160190 PMC9493365

[ref20] Kanaan M. (2023). Prevalence and antimicrobial resistance of *Salmonella enterica* serovars Enteritidis and typhimurium isolated from retail chicken meat in Wasit markets, Iraq. Vet. World. 16, 455–463. doi: 10.14202/vetworld.2023.455-463, PMID: 37041841 PMC10082727

[ref21] Kang X. Wang M. Meng C. Li A. Jiao X. Pan Z. (2022). Prevalence and whole-genome sequencing analysis of *Salmonella* reveal its spread along the duck production chain. Poult. Sci. 101:101993. doi: 10.1016/j.psj.2022.101993, PMID: 35839552 PMC9289855

[ref22] Keefer A. B. Xiaoli L. M'ikanatha N. M. Yao K. Hoffmann M. Dudley E. G. (2019). Retrospective whole-genome sequencing analysis distinguished PFGE and drug-resistance-matched retail meat and clinical *Salmonella* isolates. Microbiology 165, 270–286. doi: 10.1099/mic.0.000768, PMID: 30672732

[ref23] Konyali D. Guzel M. Soyer Y. (2023). Genomic characterization of *Salmonella enterica* resistant to cephalosporin, quinolones, and macrolides. Curr. Microbiol. 80:344. doi: 10.1007/s00284-023-03458-y, PMID: 37725171

[ref24] Ksibi B. Ktari S. Ghedira K. Othman H. Maalej S. Mnif B.. (2022). Antimicrobial resistance genes, virulence markers and prophage sequences in *Salmonella enterica* serovar Enteritidis isolated in Tunisia using whole genome sequencing. Curr Res Microb Sci. 3:100151. doi: 10.1016/j.crmicr.2022.100151, PMID: 35909609 PMC9325895

[ref25] Lamers A. P. Keithly M. E. Kim K. Cook P. D. Stec D. F. Hines K. M.. (2012). Synthesis of bacillithiol and the catalytic selectivity of FosB-type fosfomycin resistance proteins. Org. Lett. 14, 5207–5209. doi: 10.1021/ol302327t, PMID: 23030527 PMC3544479

[ref26] Li C. Zhang Z. Xu X. He S. Zhao X. Cui Y.. (2021). Molecular characterization of cephalosporin-resistant *Salmonella enteritidis* ST11 isolates carrying *Bla*(CTX-M) from children with diarrhea. Foodborne Pathog. Dis. 18, 702–711. doi: 10.1089/fpd.2020.2878, PMID: 33534635

[ref27] Li Y. Kang X. Ed-Dra A. Zhou X. Jia C. Müller A.. (2022). Genome-based assessment of antimicrobial resistance and virulence potential of isolates of non-Pullorum/Gallinarum *Salmonella* serovars recovered from dead poultry in China. Microbiol. Spectr. 10:e0096522. doi: 10.1128/spectrum.00965-22, PMID: 35727054 PMC9431532

[ref28] Liang C. Wei Y. Wang X. Gao J. Cui H. Zhang C.. (2023). Analysis of resistance gene diversity in the intestinal microbiome of broilers from two types of broiler garms in Hebei province, China. Antibiotics 12:1664. doi: 10.3390/antibiotics12121664, PMID: 38136698 PMC10741226

[ref29] Liu B. Zhou X. Zhang L. Liu W. Dan X. Shi C.. (2012). Development of a novel multiplex PCR assay for the identification of *Salmonella enterica* typhimurium and Enteritidis. Food Control 27, 87–93. doi: 10.1016/j.foodcont.2012.01.062

[ref30] Long L. You L. Wang D. Wang M. Wang J. Bai G.. (2022). Highly prevalent MDR, frequently carrying virulence genes and antimicrobial resistance genes in *Salmonella enterica* serovar 4,[5],12: i: - isolates from Guizhou province, China. PLoS One. 17:e0266443. doi: 10.1371/journal.pone.0266443, PMID: 35588421 PMC9119451

[ref31] Ma J. An N. Li W. Liu M. Li S. (2022). Antimicrobial resistance and molecular characterization of gene cassettes from class 1 integrons in *Salmonella* strains. J. Med. Microbiol. 71:001574. doi: 10.1099/jmm.0.001574, PMID: 36069773

[ref32] Ma Y. Li M. Xu X. Fu Y. Xiong Z. Zhang L.. (2018). High-levels of resistance to quinolone and cephalosporin antibiotics in MDR-ACSSuT *Salmonella enterica* serovar Enteritidis mainly isolated from patients and foods in Shanghai, China. Int. J. Food Microbiol. 286, 190–196. doi: 10.1016/j.ijfoodmicro.2018.09.022, PMID: 30268051

[ref33] Meng X. Zhang Z. Li K. Wang Y. Xia X. Wang X.. (2017). Antibiotic susceptibility and molecular screening of class I integron in *Salmonella* isolates recovered from retail raw chicken carcasses in China. Microb. Drug Resist. 23, 230–235. doi: 10.1089/mdr.2015.0359, PMID: 27309257

[ref34] Pearce M. E. Alikhan N. F. Dallman T. J. Zhou Z. Grant K. Maiden M. C. J. (2018). Comparative analysis of core genome MLST and SNP typing within a European *Salmonella* serovar Enteritidis outbreak. Int. J. Food Microbiol. 274, 1–11. doi: 10.1016/j.ijfoodmicro.2018.02.023, PMID: 29574242 PMC5899760

[ref35] Petrin S. Orsini M. Massaro A. Olsen J. Barco L. Losasso C. (2023). Phenotypic and genotypic antimicrobial resistance correlation and plasmid characterization in *Salmonella* spp. isolates from Italy reveal high heterogeneity among serovars. Front. Public Health 11:1221351. doi: 10.3389/fpubh.2023.1221351, PMID: 37744490 PMC10513437

[ref36] Qin X. Yang M. Cai H. Liu Y. Gorris L. Aslam M. Z.. (2022). Antibiotic resistance of *Salmonella Typhimurium* monophasic variant 1,4,[5],12: i: - in China: a systematic review and meta-analysis. Antibiotics. 11:532. doi: 10.3390/antibiotics11040532, PMID: 35453283 PMC9031511

[ref37] Qu M. Lv B. Zhang X. Yan H. Huang Y. Qian H.. (2016). Prevalence and antibiotic resistance of bacterial pathogens isolated from childhood diarrhea in Beijing, China (2010-2014). Gut Pathog. 8:31. doi: 10.1186/s13099-016-0116-2, PMID: 27303446 PMC4906916

[ref38] Rakitin A. L. Yushina Y. K. Zaiko E. V. Bataeva D. S. Kuznetsova O. A. Semenova A. A.. (2021). Evaluation of antibiotic resistance of *Salmonella* serotypes and whole-genome sequencing of multi-resistant strains isolated from food products in Russia. Antibiotics 11:1. doi: 10.3390/antibiotics11010001, PMID: 35052878 PMC8773070

[ref39] Rincón-Gamboa S. M. Poutou-Piñales R. A. Carrascal-Camacho A. K. (2021). Antimicrobial resistance of non-typhoid *Salmonella* in meat and meat products. Food Secur. 10:1731. doi: 10.3390/foods10081731, PMID: 34441509 PMC8392175

[ref40] Rounds J. M. Taylor A. J. Eikmeier D. Nichols M. M. Lappi V. Wirth S. E.. (2020). Prospective *Salmonella enteritidis* surveillance and outbreak detection using whole genome sequencing, Minnesota 2015-2017. Epidemiol. Infect. 148:e254. doi: 10.1017/S0950268820001272, PMID: 32539900 PMC7689598

[ref41] Silva C. Puente J. L. Calva E. (2017). *Salmonella* virulence plasmid: pathogenesis and ecology. Pathog. Dis. 75:ftx070. doi: 10.1093/femspd/ftx070, PMID: 28645187

[ref42] Song Y. Wang F. Liu Y. Song Y. Zhang L. Zhang F.. (2020). Occurrence and characterization of *Salmonella* isolated from chicken breeder flocks in nine Chinese provinces. Front. Vet. Sci. 7:479. doi: 10.3389/fvets.2020.00479, PMID: 32903795 PMC7438879

[ref43] Srednik M. E. Morningstar-Shaw B. R. Hicks J. A. Mackie T. A. Schlater L. K. (2022). Antimicrobial resistance and genomic characterization of *Salmonella enterica* serovar Senftenberg isolates in production animals from the United States. Front. Microbiol. 13:979790. doi: 10.3389/fmicb.2022.979790, PMID: 36406424 PMC9668867

[ref44] Stenfors Arnesen L. P. Fagerlund A. Granum P. E. (2008). From soil to gut: *Bacillus cereus* and its food poisoning toxins. FEMS Microbiol. Rev. 32, 579–606. doi: 10.1111/j.1574-6976.2008.00112.x, PMID: 18422617

[ref45] Sun H. Wan Y. Du P. Bai L. (2020). The epidemiology of monophasic *Salmonella Typhimurium*. Foodborne Pathog. Dis. 17, 87–97. doi: 10.1089/fpd.2019.2676, PMID: 31532231

[ref46] Sun R. Y. Fang L. X. Dai J. J. Chen K. C. Ke B. X. Sun J.. (2024). Antimicrobial resistance and population genomics of emerging multidrug-resistant *Salmonella* 4,[5], 12: i: - in Guangdong, China. mSystems 9:e0116423. doi: 10.1128/msystems.01164-23, PMID: 38747582 PMC11237462

[ref47] Takahata S. Ida T. Hiraishi T. Sakakibara S. Maebashi K. Terada S.. (2010). Molecular mechanisms of fosfomycin resistance in clinical isolates of *Escherichia coli*. Int. J. Antimicrob. Agents 35, 333–337. doi: 10.1016/j.ijantimicag.2009.11.011, PMID: 20071153

[ref48] Urrutia M. I Fuentes J. A. Valenzuela L. M. Ortega A. P. Hidalgo A. A. Mora G. C. (2014). *Salmonella Typhi shdA*: pseudogene or allelic variant? Infect. Genet. Evol. 26, 146–152. doi: 10.1016/j.meegid.2014.05.013, PMID: 24859062

[ref49] Villa L. García-Fernández A. Fortini D. Carattoli A. (2010). Replicon sequence typing of IncF plasmids carrying virulence and resistance determinants. J. Antimicrob. Chemother. 65, 2518–2529. doi: 10.1093/jac/dkq347, PMID: 20935300

[ref50] Wang W. Zhao L. Hu Y. Dottorini T. Fanning S. Xu J.. (2020). Epidemiological study on prevalence, serovar diversity, multidrug resistance, and CTX-M-type extended-spectrum β-lactamases of *Salmonella* spp. from patients with diarrhea, food of animal origin, and pets in several provinces of China. Antimicrob. Agents Chemother. 64, e00092–e00020. doi: 10.1128/AAC.00092-20, PMID: 32312775 PMC7318004

[ref51] Wang Y. Liu Y. Lyu N. Li Z. Ma S. Cao D.. (2022). The temporal dynamics of antimicrobial-resistant *Salmonella enterica* and predominant serovars in China. Natl. Sci. Rev. 10:nwac269. doi: 10.1093/nsr/nwac269, PMID: 37035020 PMC10076184

[ref52] Wottlin L. R. Harvey R. B. Norman K. N. Burciaga S. Loneragan G. H. Droleskey R. E.. (2022). Prevalence and antimicrobial resistance of nontyphoidal *Salmonella enterica* from head meat and trim for ground product at pork processing facilities. J. Food Prot. 85, 1008–1016. doi: 10.4315/JFP-22-049, PMID: 35499403

[ref53] Xu J. Sangthong R. McNeil E. Tang R. Chongsuvivatwong V. (2020). Antibiotic use in chicken farms in northwestern China. Antimicrob. Resist. Infect. Control 9:10. doi: 10.1186/s13756-019-0672-6, PMID: 31921416 PMC6947973

[ref54] Yang C. Chen K. Ye L. Heng H. Yang X. Wai-Chi Chan E.. (2023). Prevalence and molecular characterization of cefotaxime-resistant *Salmonella* strains recovered from retail meat samples in Shenzhen, China, during 2014-2017. Microbiol Spectr. 11:e0488622. doi: 10.1128/spectrum.04886-22, PMID: 37615439 PMC10580925

[ref55] Yang J. Zhang Z. Zhou X. Cui Y. Shi C. Shi X. (2020). Prevalence and characterization of antimicrobial resistance in *Salmonella enterica* isolates from retail foods in Shanghai, China. Foodborne Pathog. Dis. 17, 35–43. doi: 10.1089/fpd.2019.2671, PMID: 31532230

[ref56] Yin X. Dudley E. G. Pinto C. N. M'ikanatha N. M. (2022). Fluoroquinolone sales in food animals and quinolone resistance in non-typhoidal *Salmonella* from retail meats: United States, 2009-2018. J. Glob. Antimicrob. Resist. 29, 163–167. doi: 10.1016/j.jgar.2022.03.005, PMID: 35288333

[ref57] Yue Y. Shen M. Liu X. Hao Q. Kang Y. Che Y.. (2022). Whole-genome sequencing-based prediction and analysis of antimicrobial resistance in *Yersinia enterocolitica* from Ningxia, China. Front. Microbiol. 13:936425. doi: 10.3389/fmicb.2022.936425, PMID: 35942314 PMC9356307

[ref58] Zakaria Z. Hassan L. Sharif Z. Ahmad N. Mohd A. R. Amir H. S.. (2022). Virulence gene profile, antimicrobial resistance and multilocus sequence typing of *Salmonella enterica* subsp. *enterica* serovar Enteritidis from chickens and chicken products. Animals 12:97. doi: 10.3390/ani12010097, PMID: 35011203 PMC8749576

[ref59] Zhao L. Liu G. Tang W. Song X. Zhao X. Wang C.. (2023). Antimicrobial resistance and genomic characteristics of *Salmonella* from broilers in Shandong Province. Front. Vet. Sci. 10:1292401. doi: 10.3389/fvets.2023.1292401, PMID: 38076566 PMC10701519

[ref60] Zheng D. Ma K. Du J. Zhou Y. Wu G. Qiao X.. (2021). Characterization of human origin *Salmonella* serovar 1,4,[5],12: i: - in eastern China, 2014 to 2018. Foodborne Pathog. Dis. 18, 790–797. doi: 10.1089/fpd.2021.0008, PMID: 34287022

[ref61] Zhou X. Xu L. Xu X. Zhu Y. Suo Y. Shi C.. (2018). Antimicrobial resistance and molecular characterization of *Salmonella enterica* serovar Enteritidis from retail chicken products in Shanghai, China. Foodborne Pathog. Dis. 15, 346–352. doi: 10.1089/fpd.2017.2387, PMID: 29847740

[ref62] Zwe Y. H. Chin S. F. Kohli G. S. Aung K. T. Yang L. Yuk H. G. (2020). Whole genome sequencing (WGS) fails to detect antimicrobial resistance (AMR) from heteroresistant subpopulation of *Salmonella enterica*. Food Microbiol. 91:103530. doi: 10.1016/j.fm.2020.103530, PMID: 32539974

