# Supplementary material for: Phenotypic and WGS-derived antibiotic resistance patterns of Salmonella Enteritidis isolates from retail meat and environment during 2014 to 2019 in China
Source: Front Microbiol. 2025 Jan 27;16:1502138. doi: 10.3389/fmicb.2025.1502138 (PMC11808041; doi:10.3389/fmicb.2025.1502138)
Supplement: Supplementary file 1 [file Table_1.docx]

**Table S1 Detailed information of 95 *Salmonella* Enteritidis isolates**

|  | | | | | | | |
| --- | --- | --- | --- | --- | --- | --- | --- |
| Strains | **Region** | | **Sample type** | | **Year** | **Resistant phenotypes** | **Others** |
|  | **Province** | **Define** | **Retail meat** | **Define** |  |  |  |
| SUMHS 240055 | Shanghai | Eastern China | Retail Duck | Retail meat | 2015 | NAL-AMP-FIS-STR |  |
| SUMHS 240056 | Shanghai | Eastern China | Retail Duck | Retail meat | 2015 | NAL-AMP-FIS-STR |  |
| SUMHS 240057 | Shanghai | Eastern China | Retail Chicken | Retail meat | 2015 | NAL-STR-TET |  |
| SUMHS 240058 | Shanghai | Eastern China | Retail Duck | Retail meat | 2015 | NAL-AMP-FIS-STR-TET |  |
| SUMHS 240059 | Shanghai | Eastern China | Retail Chicken | Retail meat | 2015 | NAL-AMP-FIS-STR-TET |  |
| SUMHS 240060 | Guangdong | Southern China | Retail Chicken | Retail meat | 2015 | NAL-AMP-FIS-STR |  |
| SUMHS 240061 | Guangdong | Southern China | Retail Chicken | Retail meat | 2015 | NAL-AMP-FIS-STR |  |
| SUMHS 240062 | Guangdong | Southern China | Retail Chicken | Retail meat | 2015 | NAL-AMP |  |
| SUMHS 240063 | Guangdong | Southern China | Retail Chicken | Retail meat | 2015 | NAL-AMP-FIS-STR-TET |  |
| SUMHS 240064 | Shanghai | Eastern China | Retail Chicken | Retail meat | 2015 | NAL-AMP-FIS-STR-TET |  |
| SUMHS 240065 | Shanghai | Eastern China | Retail Chicken | Retail meat | 2015 | NAL-AMP-FIS-STR |  |
| SUMHS 240066 | Shanghai | Eastern China | Retail Chicken | Retail meat | 2015 | NAL-AMP |  |
| SUMHS 240067 | Shanghai | Eastern China | Retail Chicken | Retail meat | 2015 | NAL-AMP-FIS-STR |  |
| SUMHS 240068 | Shanghai | Eastern China | Retail Chicken | Retail meat | 2015 | NAL-AMP |  |
| SUMHS 240069 | Shanghai | Eastern China | Retail Chicken | Retail meat | 2015 | NAL-AMP-FIS-STR |  |
| SUMHS 240070 | Shanghai | Eastern China | Retail Chicken | Retail meat | 2015 | AMP-NAL-FIS | monophasic bacteria |
| SUMHS 240071 | Shanghai | Eastern China | Retail Chicken | Retail meat | 2015 | NAL-FIS |  |
| SUMHS 240072 | Shanghai | Eastern China | Retail Chicken | Retail meat | 2015 | NAL |  |
| SUMHS 240073 | Shanghai | Eastern China | Retail Chicken | Retail meat | 2015 | NAL-AMP |  |
| SUMHS 240074 | Shanghai | Eastern China | Ready-to-eat product | Ready-to-eat product | 2016 | NAL-AMP-FIS-STR-TET |  |
| SUMHS 240075 | Shanghai | Eastern China | Retail Chicken | Retail meat | 2016 | NAL-AMP |  |
| SUMHS 240076 | Henan | Central China | Retail Chicken | Retail meat | 2015 | NAL-AMP-FIS-STR |  |
| SUMHS 240077 | Henan | Central China | Retail Chicken | Retail meat | 2015 | NAL-AMP-FIS-STR |  |
| SUMHS 240078 | Henan | Central China | Freshwater fish | Freshwater fish | 2015 | NAL-AMP-FIS-STR |  |
| SUMHS 240079 | Guangdong | Southern China | Retail Chicken | Retail meat | 2016 | NAL-AMP-FIS-STR-TET |  |
| SUMHS 240080 | Shanghai | Eastern China | Retail Chicken | Retail meat | 2016 | NAL-AMP-FIS-STR-TET |  |
| SUMHS 240081 | Shanghai | Eastern China | Retail Duck | Retail meat | 2016 | AMP-NAL-FOS-STR-FIS |  |
| SUMHS 240082 | Sichuan | Southwestern China | Egg | Egg | 2014 | AMP-NAL-FOS-STR-FIS |  |
| SUMHS 240083 | Sichuan | Southwestern China | Egg | Egg | 2014 | NAL-AMP |  |
| SUMHS 240084 | Sichuan | Southwestern China | Retail Chicken | Retail meat | 2014 | NAL-FIS |  |
| SUMHS 240085 | Guangdong | Southern China | Retail Chicken | Retail meat | 2016 | NAL-AMP-FIS-STR |  |
| SUMHS 240086 | Shanghai | Eastern China | Retail Duck | Retail meat | 2016 | NAL-AMP-FIS-STR-TET |  |
| SUMHS 240087 | Shanghai | Eastern China | Retail Chicken | Retail meat | 2016 | NAL-AMP |  |
| SUMHS 240088 | Shanghai | Eastern China | Retail Duck | Retail meat | 2016 | NAL-AMP-FIS-STR-TET |  |
| SUMHS 240090 | Guangdong | Southern China | Retail Chicken | Retail meat | 2016 | NAL-STR-FIS |  |
| SUMHS 240091 | Guangdong | Southern China | Retail Chicken | Retail meat | 2016 | NAL |  |
| SUMHS 240092 | Guangdong | Southern China | Retail Chicken | Retail meat | 2016 | NAL-AMP-STR-KAN-GEN |  |
| SUMHS 240095 | Shandong | Eastern China | Retail Chicken | Retail meat | 2016 | NAL-FOS | monophasic bacteria |
| SUMHS 240096 | Shandong | Eastern China | Retail Chicken | Retail meat | 2017 | NAL-AMP-FIS-STR-TET |  |
| SUMHS 240097 | Shandong | Eastern China | Retail Chicken | Retail meat | 2017 | NAL-KAN-STR | monophasic bacteria |
| SUMHS 240098 | Shandong | Eastern China | Retail Chicken | Retail meat | 2017 | NAL-AMP-FIS-STR-TET |  |
| SUMHS 240099 | Shandong | Eastern China | Retail Chicken | Retail meat | 2017 | NAL-AMP-FIS-STR-TET |  |
| SUMHS 240100 | Shandong | Eastern China | Retail Chicken | Retail meat | 2017 | NAL-AMP-FIS-STR-TET |  |
| SUMHS 240101 | Shandong | Eastern China | Retail Chicken | Retail meat | 2017 | NAL-AMP-FIS-STR-TET |  |
| SUMHS 240103 | Shandong | Eastern China | Retail Chicken | Retail meat | 2017 | NAL-AMP-FIS-STR-TET |  |
| SUMHS 240104 | Shandong | Eastern China | Retail Chicken | Retail meat | 2016 | NAL-AMP-FIS-STR |  |
| SUMHS 240105 | Shandong | Eastern China | Retail Duck | Retail meat | 2016 | NAL-AMP-FIS-STR-TET |  |
| SUMHS 240106 | Shandong | Eastern China | Retail Chicken | Retail meat | 2016 |  | monophasic bacteria |
| SUMHS 240107 | Guangdong | Southern China | Retail Chicken | Retail meat | 2017 | AMP-STR-FIS |  |
| SUMHS 240108 | Guangdong | Southern China | Retail Chicken | Retail meat | 2017 | NAL |  |
| SUMHS 240109 | Guangdong | Southern China | Retail Chicken | Retail meat | 2017 | NAL |  |
| SUMHS 240110 | Guangdong | Southern China | Retail pork | Retail meat | 2017 | NAL |  |
| SUMHS 240111 | Shanghai | Eastern China | Retail Duck | Retail meat | 2017 | NAL-AMP-FIS-STR-TET |  |
| SUMHS 240112 | Shanghai | Eastern China | Retail Chicken | Retail meat | 2017 | NAL |  |
| SUMHS 240113 | Beijing | Northern China | Retail pork | Retail meat | 2017 | NAL-AMP-FIS-STR-TET |  |
| SUMHS 240114 | Guangxi | Southern China | Water environment | Water environment | 2018 |  | monophasic bacteria |
| SUMHS 240115 | Guangdong | Southern China | Retail Chicken | Retail meat | 2018 | NAL-AMP-FIS-STR |  |
| SUMHS 240116 | Guangdong | Southern China | Retail Chicken | Retail meat | 2018 | NAL-AMP-FIS-STR |  |
| SUMHS 240117 | Guangdong | Southern China | Retail Chicken | Retail meat | 2018 | NAL-AMP-FIS-STR |  |
| SUMHS 240118 | Guangdong | Southern China | Retail pork | Retail meat | 2018 | NAL-AMP-FIS-STR |  |
| SUMHS 240119 | Guangdong | Southern China | Retail Chicken | Retail meat | 2018 | NAL-AMP |  |
| SUMHS 240120 | Guangdong | Southern China | Retail Chicken | Retail meat | 2018 | NAL |  |
| SUMHS 240121 | Guangdong | Southern China | Retail Chicken | Retail meat | 2018 | NAL-AMP-FIS-STR-TET |  |
| SUMHS 240122 | Guangdong | Southern China | Retail Chicken | Retail meat | 2018 | NAL-AMP-FIS-STR |  |
| SUMHS 240123 | Shandong | Eastern China | Retail Chicken | Retail meat | 2018 | NAL-AMP- FIS |  |
| SUMHS 240124 | Shandong | Eastern China | Retail Chicken | Retail meat | 2018 | NAL-AMP-FIS-STR |  |
| SUMHS 240125 | Shandong | Eastern China | Retail Chicken | Retail meat | 2018 | NAL |  |
| SUMHS 240126 | Guangxi | Southern China | Water environment | Water environment | 2018 | NAL-AMP-FIS-STR |  |
| SUMHS 240127 | Hubei | Central China | Retail pork | Retail meat | 2018 | NAL-AMP-FIS-STR-TET |  |
| SUMHS 240128 | Heilongjiang | Northeastern China | Freshwater fish | Freshwater fish | 2018 | NAL-AMP-FIS-STR |  |
| SUMHS 240129 | Shanxi | Northern China | Egg | Egg | 2018 | AMP |  |
| SUMHS 240130 | Shanxi | Northern China | Egg | Egg | 2018 | NAL |  |
| SUMHS 240131 | Shanxi | Northern China | Egg | Egg | 2018 | NAL-AMP-FIS-STR |  |
| SUMHS 240132 | Shanghai | Eastern China | Retail Chicken | Retail meat | 2018 | NAL-AMP-FIS-STR |  |
| SUMHS 240133 | Shanghai | Eastern China | Retail Chicken | Retail meat | 2018 | NAL-AMP-FIS-STR-TET |  |
| SUMHS 240134 | Guangdong | Southern China | Retail Duck | Retail meat | 2018 | AMP-NAL-FIS |  |
| SUMHS 240135 | Guangdong | Southern China | Retail Chicken | Retail meat | 2019 | NAL-FIS |  |
| SUMHS 240136 | Guangdong | Southern China | Retail Chicken | Retail meat | 2019 | NAL-AMP-FIS-STR |  |
| SUMHS 240137 | Guangdong | Southern China | Retail Chicken | Retail meat | 2019 | NAL-AMP-FIS-STR |  |
| SUMHS 240138 | Guangdong | Southern China | Retail Chicken | Retail meat | 2019 | NAL-FEP-TET |  |
| SUMHS 240139 | Guangdong | Southern China | Retail Chicken | Retail meat | 2019 | NAL-AMP-FIS-STR |  |
| SUMHS 240140 | Shanghai | Eastern China | Retail Chicken | Retail meat | 2019 | NAL-AMP-FIS-STR |  |
| SUMHS 240141 | Guangdong | Southern China | Retail Chicken | Retail meat | 2019 | NAL-AMP-FIS-STR |  |
| SUMHS 240142 | Shanghai | Eastern China | Retail Duck | Retail meat | 2019 | NAL-AMP-FIS-STR |  |
| SUMHS 240143 | Guangdong | Southern China | Retail Duck | Retail meat | 2019 | NAL-AMP-FIS-STR |  |
| SUMHS 240144 | Guangdong | Southern China | Retail Chicken | Retail meat | 2019 | NAL-AMP-FIS-STR |  |
| SUMHS 240145 | Guangdong | Southern China | Retail Chicken | Retail meat | 2019 | NAL-AMP-FIS-STR-TET |  |
| SUMHS 240146 | Guangdong | Southern China | Retail Chicken | Retail meat | 2019 | NAL-AMP-TET-CRO |  |
| SUMHS 240147 | Shanghai | Eastern China | Retail Chicken | Retail meat | 2019 | NAL-AMP-FIS-STR-TET -KAN-FEP-CHL | monophasic bacteria |
| SUMHS 240148 | Guangdong | Southern China | Retail Duck | Retail meat | 2019 | NAL-AMP-FIS-STR |  |
| SUMHS 240149 | Guangdong | Southern China | Retail Chicken | Retail meat | 2019 | NAL-AMP-FIS-STR |  |
| SUMHS 240150 | Shanghai | Eastern China | Retail Duck | Retail meat | 2019 | NAL |  |
| SUMHS 240151 | Shanghai | Eastern China | Retail Chicken | Retail meat | 2019 | NAL-AMP-FIS-STR-TET |  |
| SUMHS 240152 | Shanghai | Eastern China | Retail Chicken | Retail meat | 2019 | NAL-AMP-FIS-STR-TET |  |
| SUMHS 240153 | Shanghai | Eastern China | Food poisoning | Food poisoning | 2019 | NAL-AMP-FIS-STR |  |
